# Low magnesium levels and prognosis in newly diagnosed diffuse large B-cell lymphoma

**DOI:** 10.1093/oncolo/oyae255

**Published:** 2024-10-08

**Authors:** Jennifer J Gile, Matthew Maurer, Gordon J Ruan, Jithma P Abeykoon, Joy R Heimgartner, Nikola A Baumann, Molly McMahon, Yi Lin, Thomas E Witzig

**Affiliations:** Division of Hematology, Department of Medicine, Mayo Clinic, Rochester, MN 55905, United States; Division of Clinical Trials and Biostatistics, Department of Quantitative Health Sciences, Mayo Clinic, Rochester, MN 55905, United States; Division of Hematology, Department of Medicine, Mayo Clinic, Rochester, MN 55905, United States; Division of Hematology, Department of Medicine, Mayo Clinic, Rochester, MN 55905, United States; Division of Endocrinology, Department of Medicine, Mayo Clinic, Rochester, MN 55905, United States; Division of Hematology, Department of Medicine, Mayo Clinic, Rochester, MN 55905, United States; Division of Endocrinology, Department of Medicine, Mayo Clinic, Rochester, MN 55905, United States; Division of Hematology, Department of Medicine, Mayo Clinic, Rochester, MN 55905, United States; Division of Hematology, Department of Medicine, Mayo Clinic, Rochester, MN 55905, United States

**Keywords:** nutrition, magnesium, survival analysis, non-Hodgkin lymphoma

## Abstract

Magnesium (Mg) is an essential element involved in cellular metabolism. We demonstrated that in patients with diffuse large B-cell lymphoma (DLBCL) undergoing autologous stem cell transplant (SCT), those with a serum Mg < 2.0 mg/dL at the time of transplant had worse outcomes. In this study, we aimed to learn the prognostic value of low serum Mg in patients with untreated DLBCL. We analyzed serum from 408 patients and tested 2 Mg cutpoints—low (<1.7 mg/dL) and low normal (<2.0 mg/dL), a range we found associated with lower survival in the SCT group. We found 3% of patients with low levels and 23% with low normal levels. Low normal serum Mg levels were associated with a higher stage at diagnosis, more extranodal involvement, higher international prognostic index score, lower overall survival (OS), and event-free survival. These data warrant testing Mg replacement to a target of >2.0 mg/dL to learn if survival can be improved.

## Teaser

Magnesium is an essential element involved in cellular metabolism. This study investigated the prognostic value of low serum magnesium in patients with newly diagnosed diffuse large B-cell lymphoma.

## Introduction

Low serum Mg is common in hospitalized patients and associated with increased all-cause mortality. Initial studies in patients with X-linked immunodeficiency with Mg defect, EBV, and neoplasia (XMEN disease) suggest a role for Mg deficiency in the development of hematologic malignancies. In these patients, a mutation in an Mg transport channel impairs Mg influx which impairs T-cell activation.^1,2^ We have previously demonstrated that serum Mg levels < 2.0 mg/dL at the time of stem cell transplant (SCT) for relapsed DLBCL is associated with inferior event-free survival (EFS) and OS posttransplant.^3^ We identified that hypomagnesemia prior to chemotherapy in patients with Burkitt lymphoma is associated with inferior OS.^4^ These findings have been confirmed in patients with low serum Mg level undergoing chimeric antigen receptor T-cell therapy, where patients were found to have more rapid disease progression and shorter OS.^5^ Given these data, in this study, we aimed to determine the incidence of serum Mg levels and prognosis in untreated DLBCL.

## Methods

This study was reviewed and approved by the Mayo Clinic Institutional Review Board (IRB). Patients eligible for the study had a confirmed diagnosis of DLBCL, available pretreatment cryopreserved serum from the biobank, and were enrolled into the IRB-approved University of Iowa/Mayo Clinic Lymphoma SPORE Molecular Epidemiology Resource between 2006 and 2015. Patients provided written, informed consent for the use of their biobank sample for research. Baseline clinical, laboratory, and treatment data were abstracted from the medical records. Serum samples from 408 patients were identified with the serum Mg level measured in the Mayo Clinic clinical laboratory. We previously had shown that serum Mg level testing on frozen samples is reliable. We tested 2 Mg cutpoints—low (<the laboratory lower limit 1.7 mg/dL) and low normal < 2.0 mg/dL, a range we had previously established to be associated with lower survival in the SCT group.^3^

The outcomes of interest were OS defined as the time from diagnosis to death from any cause and EFS defined as the time from diagnosis to progression, initiation of a second-line therapy, or death from any cause. OS and EFS were analyzed using the Kaplan-Meier method. Cox regression models were used to calculate hazard ratios (HR) and 95% CIs. Additionally, a Pearson’s chi-squared test was used to examine differences in clinical characteristics stratified by Mg levels. Statistical analyses were performed using R (version 4.1.2). An unadjusted *P*-value of <.05 was considered statistically significant.

## Results and Discussion

The characteristics of the 408 patients are summarized in Table 1. Twenty-three percent (94/408) of patients had a serum Mg level < 2.0 mg/dL, and only 3% (14/408) had a serum Mg level < 1.7 mg/dL. Low normal serum Mg levels were associated with advanced stage at diagnosis (*P* = .027), increased extranodal involvement (*P* < .001), and increased international prognostic index (IPI) score (*P* = .006).

**Table 1.** Demographic and clinical correlates of magnesium levels.

Variables	All patients (*N* = 408)	Mg < 2 (*N* = 95)	Mg ≥ 2 (*N* = 313)	*P*-value
Age at diagnosis, median (range)	64 (18–90)	65 (19–90)	63 (18–89)	.383
Gender (%)				
Male	57	58	56	.817
Female	43	42	44	
Stage at diagnosis (%)				
1-2	44.6	34.7	47.6	.027
3-4	55.4	65.3	52.4	
LDH (%) Elevated	49.0	56.8	46.6	.092
ECOG PS (%) ≥2	6.6	7.4	6.4	.737
Extranodal involvement (%) >1	25.2	40.4	20.6	<.001
IPI (%)				
Low	38.2	26.2	41.9	.006
Low intermediate	28.2	27.4	28.4	
High intermediate	23.8	29.5	22.0	
High	9.8	16.8	7.7	
EFS 24 (%) Achieved	75.0	68.1	77.1	.07

LDH, lactate dehydrogenase; ECOG PS, Eastern Cooperative Oncology Group performance status; IPI, international prognostic index; EFS, event-free survival; OS, overall survival.

**Figure 1.**
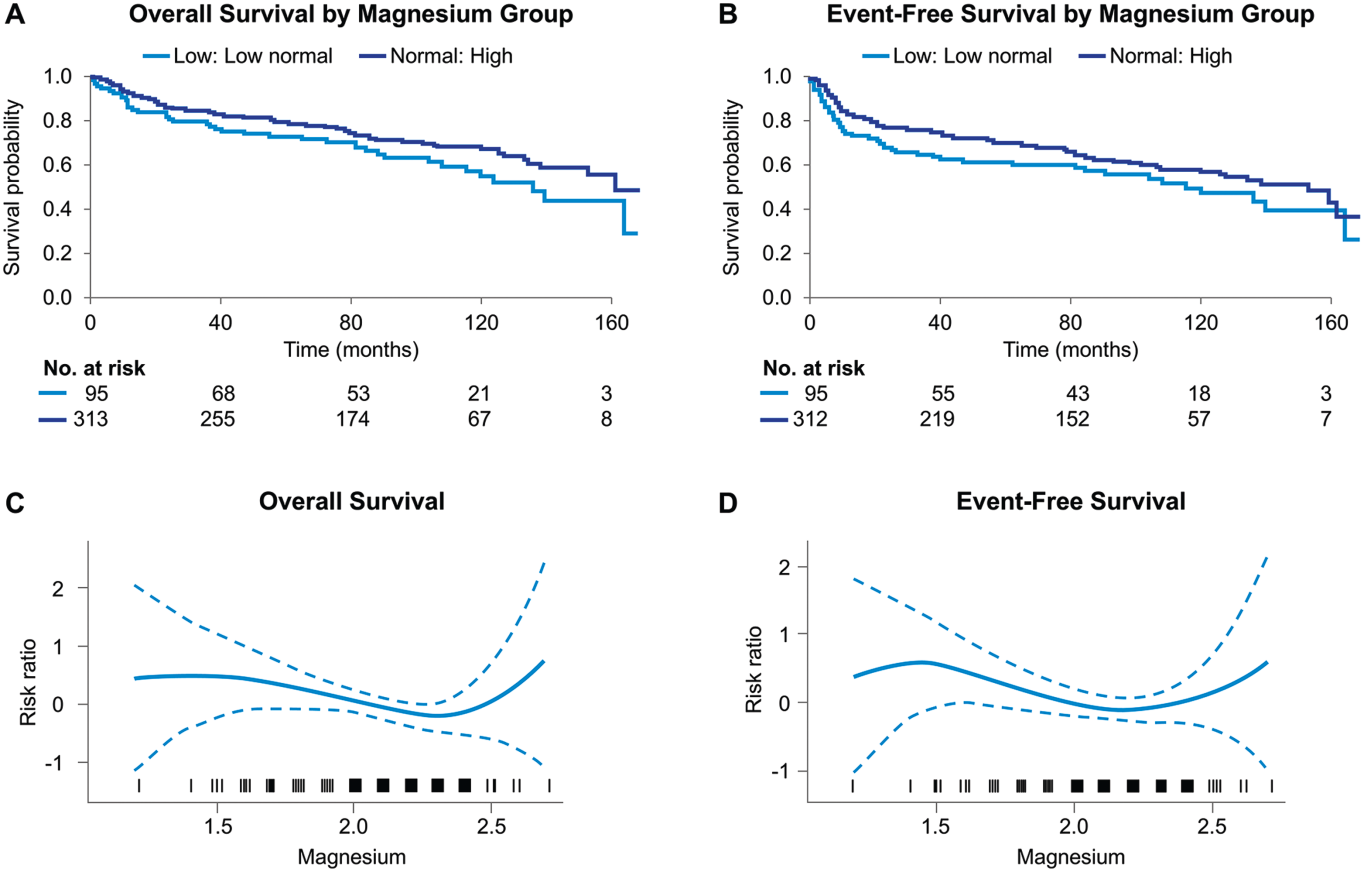
(A) Overall survival of patients with diffuse large B-cell lymphoma by pretherapy serum magnesium levels using a ≥2.0 mg/dL cutoff. Log-rank test *P* = .071. (B) Event-free survival (EFS) of patients with diffuse large B-cell lymphoma by pretherapy serum magnesium levels using a ≥2.0 mg/dL cutoff. Log-rank test *P* = .094. Log-scale hazard of the functional form for the association of (C) overall survival and (D) EFS by serum magnesium level (mg/dL) prior to treatment with chemotherapeutics supporting the ≥2.0 mg/dL target concentration.

The median follow-up was 8.1 years, with 169 events and 136 deaths. Median OS in patients with low normal (< 2.0) serum Mg levels was 11.2 years compared to 13.3 years in patients with high normal (≥ 2.0) serum Mg levels (HR = 1.40, 95% CI, 0.97-2.03, *P* = .072). The median EFS in patients with low normal serum Mg levels was 9.6 years compared to 12.6 years in patients with high normal serum Mg levels (HR = 1.33, 95% CI, 0.95-1.87, *P* = .094; Figure 1). The EFS at 24 months (EFS24) is a key prognostic metric in DLBCL.^6^ Sixty-eight percent of patients with low normal serum Mg levels achieved EFS24 compared to 77.1% of patients with high normal serum Mg levels (*P* = .07). The inferior EFS, EFS24, and OS in patients with low normal serum Mg levels indicate a potential role for Mg not only in the risk of relapse of the lymphoma but also may reflect more comorbidities in these patients. We chose a target Mg level of ≥2.0 mg/dL based on our previous work in patients with lymphoma undergoing SCT^3^ and the previous studies in cardiology showing that a serum Mg level ≥ 2.0 mg/dL is associated with improved outcomes.^7^

Although we have previously shown that low serum Mg levels in patients with DLBCL prior to undergoing SCT^3^ and in patients with Burkitt lymphoma^4^ had inferior outcomes and that low serum Mg is an independent prognostic factor for poor outcomes, it was only a trend in this study. We found that only 3% of patients had serum Mg levels below the lab normal range of 1.7-2.3 mg/dL compared to 14% in the pretransplant setting in our previous study.^3^ This likely reflects that in this study the patients were untreated whereas the SCT trial had patients that were previously exposed to chemotherapeutic agents.^8^

We demonstrated that low serum Mg levels are correlated with a more advanced IPI, a known predictor of poor outcomes in patients with DLBCL. While IPI cannot be manipulated, Mg replacement is a readily available intervention and may offer benefits to patients with newly diagnosed DLBCL. Based on previous studies that Mg is an important element for T-cell function, we hypothesize that low serum Mg levels prior to initiation of therapy may impair T-cell function and lead to a decrease in EFS/OS. Limitations of our study are that we did not have serial measurements of Mg, diet history, or information on cardiac arrhythmias in the patients. A strength of our study is that we had a long follow-up time as we recruited patients from 2006 to 2015, however, a limitation as a result of this is that we do not have information regarding how many patients were double or triple hit as FISH was not reliably done in this era, a known poor prognostic factor for patients with aggressive lymphoma. A key remaining question is whether Mg replacement or supplementation to Mg ≥ 2.0 in patients with newly diagnosed DLBCL and suboptimal Mg levels will lead to improved outcomes is unknown but warrants further investigation. Indeed, these data provide a rationale for prospective trials of Mg replacement to a level ≥ 2.0 mg/dL in patients with DLBCL starting at the time of diagnosis.

## Data Availability

The data underlying this article will be shared on reasonable request to the corresponding author.
